# “My midwife said that having a glass of red wine was actually better for the baby”: a focus group study of women and their partner’s knowledge and experiences relating to alcohol consumption in pregnancy

**DOI:** 10.1186/s12884-015-0506-3

**Published:** 2015-04-01

**Authors:** Fiona Crawford-Williams, Mary Steen, Adrian Esterman, Andrea Fielder, Antonina Mikocka-Walus

**Affiliations:** School of Nursing and Midwifery, University of South Australia, City East Campus, Frome Road, Adelaide, Australia; Department of Health Sciences, University of York, Seebohm Rowntree Building, Heslington York, UK

**Keywords:** Pregnancy, Alcohol, Foetal alcohol spectrum disorders, Health education

## Abstract

**Background:**

While it is well established that alcohol can cross the placenta to the foetus and can affect an infant’s development, many women continue to drink during pregnancy. For this reason it is important to determine what information is being provided, what information may be missing, and the preferred sources of information on this issue. In order to improve prevention strategies, we sought to understand the knowledge and experiences of pregnant women and their partners regarding the effects of alcohol consumption during pregnancy.

**Methods:**

The current study utilised a qualitative study design in order to gain insight into the views and experiences of pregnant women, newly delivered mothers and their partners. Focus groups examined the participant’s knowledge about the effects of alcohol consumption during pregnancy, the sources of information on this issue, and the psycho-social influences on their drinking behaviour. Five focus groups were conducted involving a total of 21 participants (17 female). A six-stage thematic analysis framework was used to analyse all focus group discussions in a systematic way**.**

**Results:**

Seven major themes were identified from the focus group data: 1) knowledge of Foetal Alcohol Spectrum Disorders; 2) message content and sources; 3) healthcare system; 4) society and culture; 5) partner role; 6) evaluation of risk; and 7) motivation. The findings indicated that although the majority of participants knew not to drink alcohol in pregnancy they had limited information on the specific harmful effects. In addition, routine enquiry and the provision of information by health care professionals were seen as lacking.

**Conclusions:**

The findings of this research provide important insights in to the relationship between pregnant women, their partners, and their healthcare providers. Several recommendations can be made on the basis of these findings. Firstly, public health messages and educational materials need to provide clear and consistent information about the effects of alcohol consumption on the developing baby. Additionally, more thorough and consistent routine enquiry for alcohol consumption in pregnant women needs to occur. Finally, it is important to ensure ongoing education for health professionals on the issue of alcohol consumption during pregnancy.

## Background

The harmful effects of alcohol consumption during pregnancy are well established [1-3]. Prenatal alcohol exposure can lead to a variety of adverse consequences, falling under the umbrella term of Foetal Alcohol Spectrum Disorders (FASD). These conditions can result in a range of physical, developmental and neurobehavioural abnormalities [4]. At the higher end of the spectrum, associated with heavy alcohol consumption or binge drinking, is Foetal Alcohol Syndrome (FAS). FAS is characterised by distinctive facial deformities, growth deficiencies, as well as developmental problems such as learning difficulties, lowered IQ and poor attention span [1,5]. While FAS may be easier to diagnose at birth due to the nature of the physical birth defects, prenatal alcohol exposure can lead to a range of developmental problems that may not be visible, and often may not be noticed until a child enters schooling [6]. There is still a significant lack of evidence surrounding the effects of low to moderate alcohol consumption during pregnancy [7,8]. Estimates of the prevalence of FAS or FASD vary between countries and ethnic groups, and have been difficult to determine due to inadequate means of consistent diagnoses [1].

Given the uncertainty and lack of evidence surrounding the effects of low amounts of alcohol consumption during pregnancy, the latest Australian guidelines recommend that for pregnant women or women planning a pregnancy, not drinking is the safest option [9]. However, due to multiple amendments in the Australian guidelines over the last two decades, differing world-wide policies [10,11] and conflicting media portrayal [12,13], it is understandable that this has led to some confusion among pregnant women and the general public about the accepted level of alcohol consumption during pregnancy, as evidenced by significant numbers of pregnant women continuing to drink during pregnancy despite the current guidelines [14]. It is therefore important, in order to reduce the incidence of FASD in Australia, to ensure that the correct and consistent information is being provided to pregnant women by all stakeholders, and that the most effective method of disseminating this information is being utilised.

The amended guidelines in Australia may also have impacted the knowledge of health professionals who routinely provide antenatal care for these women. Recent research has reported that health professionals were unlikely to ask pregnant women about their alcohol consumption, with some believing that these women already knew not to drink alcohol [15]. It was also reported that many health professionals assume that pregnant women are aware of the risks of alcohol consumption during pregnancy [15]. A survey of health professionals in Western Australia reported only 45% (n = 659) routinely ask about alcohol use during pregnancy, and only 25% (n = 659) routinely provide information about the effects of alcohol consumption on the foetus [16].

Furthermore, previous studies investigating the knowledge and attitudes of pregnant women towards alcohol consumption have more often than not failed to include the views of the women’s partners. Expectant fathers may play a key role in healthy pregnancy outcomes, especially with regards to alcohol consumption, since women’s alcohol use is often influenced and encouraged by other people, including partners [17]. A study conducted in 2005 included the women’s partners in a single session brief intervention aimed at reducing alcohol use among pregnant women [18]. This intervention was given both to pregnant women who had screened positive for alcohol use, in addition to the women’s partner (including spouse, father of the child, or any other supportive adult). The results of this research indicated that the intervention was more effective for women whose partner was involved than for women participating on their own [18]. Recent Australian research reported that 38% of women (n = 1103) would be less likely to drink alcohol if their partner or spouse encouraged them to cut back or stop drinking during the pregnancy [19]. This is in line with research undertaken in Canada which involved a sample of 902 women and suggested that almost 40% (n = 902) of women would be less likely to consume alcohol if they were encouraged to stop by their partner [20]. It is, therefore, clear that a women’s partner may play a significant role in reducing alcohol consumption during pregnancy, therefore it is important to explore and understand how much partners know about the issue.

### Aim and objectives

The aim of this research was to identify gaps in knowledge about the effects of alcohol use in pregnancy among pregnant women, newly delivered and their partners. The objectives were to determine the sources of their information, the quality of information provided, as well as the influence of friends, family, and partners on their drinking habits. It is envisaged that this information will be useful in improving future public health messages in order to clarify the information that is provided to pregnant women in Australia. Further, this information may be used to improve communication between health professionals, women, partners and families.

## Methods

### Design

The current study utilised a qualitative study design in order to gain a deeper insight into the views and experiences of the target population. Focus groups have increasingly been used in health research as they are a valuable method of exploring complex behaviours and motivations [21]. Focus groups were chosen over one-on-one interviews to give participants an opportunity to use the responses of others to stimulate discussion. The focus group format allowed for an in-depth examination of the issues, along with the opportunity for women and their partners to guide the development of future interventions and health services that meet consumer needs [21]. The procedure involved in the focus groups has been gathered from Elliot and associates [22] ‘Guidelines for Conducting a Focus Group’. Despite conflicting suggestions for the ideal number of participants in a focus group, many authors suggest that an adequate group size is from 4 to 12 participants, with the optimal size being between 5 and 10. However, it is argued that smaller groups can be effective for complex topics [21].

### Participants

A total of five focus groups were conducted with groups ranging from two to seven participants (see Table 1). A sample of convenience was recruited in order to obtain a wide variety of responses from participants. Flyers were placed at the Women’s and Children’s Hospital and University of South Australia, and interested participants were requested to make contact with the researcher via email. In total, 21 individuals participated in focus group discussions out of 30 people who expressed interest in the study. Due to the nature of the sample, participants were welcome to bring children along to the focus groups if necessary; this occurred in three of the focus groups.

**Table 1:** **Characteristics of focus groups**

**Focus group date**	**Number of participants**	**Structure and characteristics of focus group**
7^th^ February 2014	4	Four pregnant women
4^th^ March 2014	5	Two pregnant women, two male partners, one mother
1^st^ April 2014	2	Two pregnant women
7^th^ May 2014	7	Six mothers, one pregnant woman
4^th^ June 2014	4	One pregnant women, one mother, two male partners

### Data collection

The focus groups were conducted from February to May 2014. The focus groups were moderated by the first author (FCW) and held in a private recreational room at the University of South Australia. A focus group guide was developed in conjunction with other researchers to ensure objectivity. This guide enabled the moderator to steer conversation in specific directions. The discussions revolved around: the adverse consequences of drinking during pregnancy; the partner’s role in health decisions; the sources of information about alcohol use in pregnancy; and the availability of reliable health information. Each focus group ranged between 90 minutes to two hours. Data saturation was reached after five focus groups were conducted.

### Data analysis

Focus groups were audio-taped and transcribed verbatim, with field notes written by the moderator at the end of each session. Transcripts were read several times and analysed using thematic analysis techniques. Thematic analysis is a method of identifying, analysing and reporting patterns within data [23]. A six-step protocol described by Braun and Clarke [23] was used to analyse all focus group discussions in a systematic way. The analysis involved deriving data extracts from field notes, summaries, and verbatim transcripts. Extracts of data were then coded into logical concepts, and these codes were categorised, re-categorised and condensed to identify the major themes. The themes and sub-themes identified through the analysis were reviewed and cross-checked with other researchers, and finally named and defined [23].

### Ethical considerations

Approval was granted by both the University of South Australia’s and the Women’s and Children’s health Network Human Research Ethics Committee prior to the study’s commencement (Protocol no. 0000031358 and HREC/13/WCHN/121 respectively). Participants were invited to take part in the research after written consent was obtained to conduct and record the focus groups. Participants were assured that anonymity and confidentiality would be upheld. The moderator and primary researcher (FCW) did not have any clinical relationships with the participants prior to recruitment which may have introduced bias. The moderator explained the goals of the research at the commencement of each focus group and interview.

## Results

### Demographic information

Focus group participants ranged in age from 23 to 40 years. The total of 21 participants included eight pregnant women (gestation 13 to 38 weeks), nine mothers who recently gave birth (infants aged between 4 and 20 weeks), and four partners of pregnant women. Eighty-one percent of the sample participants were female. The majority of participants were Caucasian (95%), with one woman of Asian descent (5%). Two participants were from New Zealand (10%) while the rest were Australian (90%).

### Major themes identified

**Figure 1:**
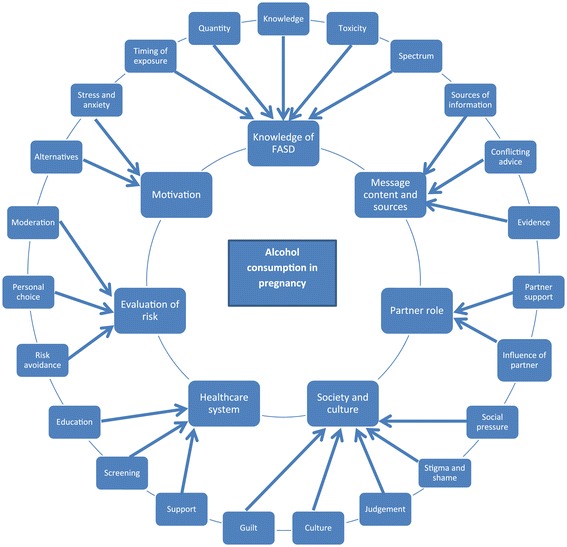
**Thematic map of codes and themes.**

Seven major themes were identified from the focus group data: 1) knowledge of FASD; 2) message content and sources; 3) healthcare system; 4) society and culture; 5) partner role; 6) evaluation of risk; and 7) motivation. Figure 1 displays the thematic map. Verbatim quotes from participants are provided to illustrate these themes.

#### Knowledge of foetal alcohol spectrum disorders

The majority of participants were aware that alcohol could cause harm to their developing baby. Although participants knew that alcohol was harmful, several participants declared that they had limited information about the actual effects. Other participants had more awareness and listed physical consequences, such as low birth weight, abnormal facial features, and poor growth as some of the more commonly known effects.

*“I generally know that you’re not meant to drink, but the exact health effects and development of the foetus…I’m not sure exactly what it affects…other than you’re not meant to drink”* (mother #1)

*“Don’t they get like a broad nose and slit eyes. Yeah if the baby gets Foetal Alcohol you can see it in their face. Like their eyes are really small and their nose is broad”* (mother #7)

*“I hadn’t heard of Foetal Alcohol Syndrome, I’ve only researched, when I did my own research I heard about just the facial deformities, physical deformities, not anything else.”* (pregnant woman #2)

*“Different levels of harm, caused by different levels of drinking, at different stages in the development of the baby. So there’s not just Foetal Alcohol Syndrome, is that sort of up on the further end of the continuum, but there’s also a range of other things that can go wrong, or happen.”* (pregnant woman #1)

Other common sub-themes that came under ‘knowledge of FASD’ included the timing of the exposure, and the quantity of alcohol consumed. It was verified by several participants that alcohol consumption in the first trimester would cause the most harm, and it was generally accepted by all participants that small amounts of alcohol, such as one or two glasses throughout the whole of pregnancy would not be harmful to the foetus.

*“I always thought you couldn’t have anything. It was sort of not ideal to conceive while you’re drunk or be drunk in the first 12 weeks or then to term, but then you see and hear lots more people who sort of think that a glass of wine is actually ok because it calms the mother’s mood which is better for the baby…”* (partner #2)

*“I’ve heard people say that as long as you don’t have it in the first 12 weeks you should be fine, like a glass here or glass there. That’s the things I’ve heard when I’ve been talking to girlfriends or something”* (pregnant woman #4)

*“I mean, if you’re at dinner and someone is pregnant and they have a sip of the wine, I don’t think anything of it, but if you’re out at a pub and then someone has a $15 cocktail that’s got 4 shots or something in it then I feel you shouldn’t”* (pregnant woman #3)

*“You just know that you shouldn’t drink, or maybe one or two is ok. I’ve heard doctors say, and I’ve read that one or two drinks will be ok”* (pregnant woman #5)

#### Message content and message sources

The sources of information stated regarding alcohol use in pregnancy ranged from health professionals such as GPs and midwives, to the internet, media, other mothers, or the woman’s own mother. It was often expressed that there was a lot of conflicting or unclear advice on the issue of alcohol use in pregnancy and that certain sources of information may be unreliable.

*“I’ve got conflicting information from different sources. So like my GP I didn’t really get a definitive answer, on you know, no drinking is the safest, that wasn’t really, sort of communicated, Whereas when I went to my midwife, for my antenatal appointment, that was clearly communicated from day one because of when I had my interview…we discussed my drinking so that came up… And then friends, I guess people do different things and that all kind of, well for me anyway, that all kind of impacts on what I’ve done, yeah. It’s not a black and white thing for me.”* (pregnant woman #1)

Several participants claimed not to have received information about alcohol use in pregnancy from their antenatal care provider. It was also felt by certain participants that health professionals often did not mention alcohol use in pregnancy as it was assumed to be common knowledge.

*“I saw both a GP and midwife but I don’t think either of them mentioned it”* (mother #2)

*“No one has really spoken to me about alcohol. I think it might have been in a pamphlet or something I got. But no one has actually had a conversation with me, and asked if I drink alcohol, during my GP and midwife check-ups”* (pregnant woman #5)

*“I can’t say that my obstetrician really mentioned not drinking or alcohol at all to me really. Maybe it was just assumed that I would know that you’re not meant to drink, so I didn’t receive any brochure about it or written information on it”* (mother #1)

Although several participants admitted to consuming small amounts of alcohol during their pregnancy, all of the participants believed that the current Australian guidelines recommended no alcohol.

*“I’ve actually heard they don’t exactly know what is safe and what isn’t. Because they don’t know they say not to do it”* (mother #6)

*“When it comes to alcohol in pregnancy my understanding is that there hasn’t been enough research, and there never will be enough research to say this is the safe level stay below it, and so for that reason they say don’t drink at all, because too much causes foetal alcohol syndrome so you don’t want to get to that point of too much, so we don’t really know where too much is”* (pregnant woman #7)

#### Healthcare system

Although the participants viewed health professionals as a trusted source of information, it appears that health professionals provide limited information regarding alcohol use in pregnancy to their patients. It was commonly expressed that women were routinely asked about their alcohol use at their first antenatal booking appointment and that this was not followed up again throughout the pregnancy.

*“When I went to the GP when I first knew I was pregnant they didn’t say anything to me about it. All they said to me was “do you know what to eat?” not any of the other stuff, it was just that I stay away from the deli counter basically. And then I had my 12 week one, where you sit with the midwife, and all they said to me was “are you smoking or drinking?” and that was it, and there was no other conversation about it”* (pregnant woman #4)

*“The midwife brought it up at my booking appointment. They ask, ‘do you drink?’ and you say no… I had to see a dietician as well, and they said straight away … you can eat this, you can’t eat that and obviously you’re not drinking at all. They don’t even say “well if you are having a drink then have only one or two”, because it’s just assumed that you don’t, you know”* (pregnant woman #8)

Some participants claimed that certain midwives had in fact endorsed drinking while pregnant, suggesting wine was okay to drink if you were craving it.

*“My midwife said that having a glass of red wine was actually better for the baby”* (mother #3)

*“There was a study that said that a glass of red wine a day was better for the baby for brain development”* (mother #6)

*“My midwife said that if you’re craving a glass of wine to have a glass”* (mother #2)

#### Society and culture

The central idea that emerged under the theme of social influences was that all the women who were noticeably pregnant or had been pregnant in the past felt that there was strong judgement and social pressure against drinking. It was reported that women felt judged most often by strangers, or people who did not know the context.

*“I wasn’t even drinking but I felt people were judging me…because I was out at night”* (mother #6)

*“You sort of think, yeah I’d love to have a glass of champers for a special occasion or something, but there’s always someone that is going to say something”* (pregnant woman #7)

*“Yeah I think there’s definitely a level of stigma, going into the bottle shop when you look like this … or going out like this. We would usually go out together to have a drink, and… as I’ve progressed through my pregnancy I’ve sort of become more aware of … how I look, so you sort of don’t feel as comfortable I guess. Even though you might drink at home, in public you sort of feel a bit scrutinised sometimes. People have pretty strong views on it, so for me, I’ve tended to go out less to have a drink, whereas I might have a drink at home.”* (pregnant woman #1)

Six participants also reported that close friends and family often encouraged the pregnant woman to drink.

*“I feel more pressure not to be around certain friends because I’m the odd one out not smoking and drinking while I’m pregnant. I don’t want to be around them”* (pregnant woman #5)

*“My mum, when we go over there, she still asks … if I would like a glass of wine. And I have to remind her that no, I’m pregnant”* (pregnant woman #4)

Another emergent idea was that culture has changed over time, and it is currently socially unacceptable for pregnant women to be seen drinking whereas women in previous generations may have drank while pregnant and not seen any harm in their children.

*“Years ago our parents and our parents’ parents weren’t told any of this, and just went along with everything they did every day in their normal life, and a lot of those kids are quite normal”* (pregnant woman #2)

#### Partner role

The notion of the partner’s role in the pregnancy was another significant theme that emerged from the data. Some partners showed support by cutting back their own drinking, while others continued to drink; however, all women indicated that they were satisfied with the support they received from their partner with regards to alcohol use during pregnancy.

*“I think my partner is probably drinking less…because I’m not drinking”* (pregnant woman #8)

*“My husband did the opposite; he said he was drinking for two”* (mother #7)

*“I always thought that when my wife gets pregnant I would stop drinking as well. Because that is kind of bit like carrying the burden, a bit like sharing. And I haven’t had a drink since she’s been pregnant, but then we didn’t drink before she was pregnant either.”* (partner #1)

Several women felt that the decision not to drink was a family decision as it would have effects on the baby, and that their partner had input on many of the health decisions made during pregnancy.

*“it’s a family decision; it’s not just about me”* (pregnant woman #7)

*“ultimately it is their (the men’s) kid…it’s your body but it’s their kid”* (mother #4)

#### Evaluation of risk

One of the codes that emerged inductively from the data under the theme of ‘evaluation of risk’ was that of personal choice. It was repeatedly mentioned in all focus groups that alcohol use in pregnancy was a personal choice, and that there was no right or wrong decision, it was up to the individual woman.

*“I did drink a bit; it was a personal choice”* (mother #4)

*“I guess it was a personal choice…I was drinking before getting pregnant then decided not to continue”* (mother #2)

*“I had IVF and it cost a hell of a lot of money so I thought I’m not doing anything wrong here”* (pregnant woman #6)

Parallel to this idea of personal choice the codes of trust and responsibility also emerged from the data. Trust implying that the pregnant woman should be trusted to make her own decisions, and responsibility implying that a pregnant woman must accept the consequences of her own actions.

*“I actually have a private midwife and she is pretty open to anything. She’s like I trust that you did your research, and do what you think is best … she sort of says you know what the risks are, so if you choose to do it you choose to do it.”* (pregnant woman #7)

This links with the idea of informed choice which emerged from the data extracts.

*“I think it is definitely a balancing act. It comes down to lots and lots of different things in pregnancy. It’s about balance, and if you are miserable because you are depriving yourself of things you love then that’s a pretty big commitment, that’s huge so I think it is about balance. If you are going to make the decision to do it, do it in an informed way, but I don’t think that one or two is bad”* (pregnant woman #7)

It was also revealed that many women felt that moderation and balance were ideal in pregnancy. As there were so many things to avoid during pregnancy it was felt necessary to create a balance as individual women struggled more in some areas than others

*“I went wrong in other ways, like soft cheese… just in my last trimester”* (mother #3)

*“I sort of figured if I wasn’t drinking any caffeine then maybe a tiny bit of wine is ok. That was my logic behind it, maybe not very good logic, but.”* (mother #1)

#### Motivation

The final theme that emerged from the focus group discussions was that of motivation. It was commonly agreed that no pregnant woman wants to cause harm to her baby by consuming alcohol, and that there must be motivations for consuming alcohol or not consuming it. The health of the developing baby was listed as the biggest motivator to change any health behaviour in pregnancy. However, stress was a key motivator for the consumption of alcohol in pregnancy, and many women agreed that if alcohol was associated with emotional benefits it would be harder to give up in pregnancy.

*“I’ve got a sister-in-law who would have 5 or 6 glasses of wine quite often, and she’s about 32 weeks pregnant. But she is a really anxious, stressed-out sort of person, so I think if you can balance out, if you are in a bad mental state or you need a drink that helps you relax I think it’s better for you to be relaxed than for you to be really tense and anxious”* (pregnant woman #8)

*“Maybe it’s a normal part of you come home and you have a glass of wine to unwind like what you can do, you and your partner, instead of doing that. Like go for a walk, play video games, I don’t know, like do things that distract you or relax you”* (pregnant woman #4)

## Discussion

This study revealed a variety of knowledge levels and experiences related to alcohol consumption during pregnancy among Australian participants. Women and their partners were found to have differing views on the risk associated with consuming alcohol, and their evaluation of risk was impaired by conflicting advice and individual differences. In addition, women often reported that they had not received targeted advice or information from health professionals.

This research has indicated that most women and their partners recognise that alcohol has the potential to cause harm to the unborn baby. However, the quantity of alcohol required to cause harm, and the impact of the timing of the exposure were not as well known. Many women could not adequately describe the effects that alcohol may have on the unborn baby, and were more likely to believe that alcohol consumption is associated with physical birth defects and facial deformities which are caused by excessive alcohol consumption. Problems associated with brain development of the foetus, such as low IQ, behavioural issues, and learning difficulties, were mentioned by some participants although these were much less commonly reported by focus group participants. While FAS may be the most serious consequence of prenatal alcohol consumption, resulting in both physical deformities and developmental issues [5], it is important that women and their partners are aware of the initially invisible damages that can occur due to prenatal alcohol consumption. These adverse consequences of alcohol consumption during pregnancy often become visible later in life as the child may act out in school or display poor learning outcomes due to lack of attention and comprehension. Furthermore, alcohol consumption during pregnancy has the potential to cause damage throughout the continuum of pregnancy, not only in the first trimester. Therefore it is again of utmost importance that pregnant women are fully aware of the effects of alcohol consumption during pregnancy at all time periods.

Almost all of the participants believed that small amounts of alcohol were unlikely to cause harm, and were acceptable in pregnancy, particularly for a special occasion, and that the majority of harmful consequences were associated with excessive or binge drinking. They were correct in acknowledging that there is a lack of evidence about the effects of small to moderate amounts of alcohol [7]; however, it is important for woman to be aware that the risk is different for each individual woman and that a small amount has the potential to cause harm depending on a wide range of contributing factors [1].

Previous research has determined that health professionals often do not ask pregnant women about their alcohol consumption as they believe that most women already know not to drink alcohol during pregnancy, or believe that the information is not relevant for the individual woman in their practice [15]. The current study highlights, that it was routine practice for health professionals to enquire about alcohol use at the initial booking appointment. However, participants’ responses clearly demonstrated that health professionals did not continue to assess the frequency and quantity of alcohol consumption at subsequent antenatal appointments. In order to ensure adequate information and support is provided to pregnant women and their partners, health professionals need to make certain that continuous enquiry about the quantity and frequency of alcohol consumption is undertaken, and to advise that drinking alcohol during the whole of pregnancy is recommended by national guidelines. Additionally, the findings of this study suggest that women and partners would like to have been given more information on why drinking in pregnancy is harmful by health professionals.

Another theme that emerged from the data and of which there is little previous research, is the issue of moderation. It was hard for several of the women in this study to avoid all of the foods and drinks that are not recommended during pregnancy, and this meant that some women would make concessions such as drinking alcohol but avoiding caffeine, or skipping both but eating soft cheese. It needs to be recognised that it is difficult for women to remove a lot of rewarding food and drink whilst pregnant, and that despite being informed of the risks of these substances, they may need help such as providing healthy alternatives (hard cheese, decaf coffee, mocktails etc.). There is limited existing research that explores the notion of moderation [24]; however, this issue has important clinical significance for health professionals who work with pregnant women and their partners, this would benefit from further exploration. Moreover, several participants felt that there was an overwhelming amount of information to process during pregnancy, particularly with regards to diet and nutrition; this was often very challenging to understand. This further highlights the need for health professionals to provide clear and concise information. This was expressed particularly by first time pregnant women, and there may be a significant difference in the information requirements needed by these expectant mothers when compared to others whom have experienced pregnancy previously.

Another interesting finding that emerged from the focus group discussions was the idea that drinking alcohol during pregnancy was a personal choice to be made by a woman. It was suggested that women would weigh up the benefits and risks and making an informed decision. The perception that it can be a personal choice conflicts with the consequences, as it is the developing baby who will be most affected by the decision and they have had no say in the decision to drink. This presents an ethical dilemma as the traditional practices of health care are based on principles of autonomy and beneficence; however, care for a pregnant woman can create a conflict between the rights of the woman and the rights of the foetus [25]. It has been suggested that the ethical responsibilities of health professionals require that the best interests of the foetus be served, and research in the US has found that 95% (n = 847) of physicians felt that pregnant woman also have a moral responsibility to ensure the health of their unborn baby [26]. On the other hand, it has also been suggested that pregnant women have the “right and responsibility to make informed decisions for herself and her foetus” [25]. The complexity of this issue highlights the need for accurate and comprehensive information about alcohol consumption in pregnancy in order for women to be empowered to make healthy decisions. Moreover, it again emphasizes the demand for effective communication between pregnant women and health professionals in order to ensure that both the mother and foetus are receiving the best healthcare possible.

Continuing on from the idea of risk evaluation is the idea that alcohol can be used to relieve stress for the mother and this might outweigh the risk of harm to the baby. Women who are feeling anxious and stressed need to be clearly informed of the potential risks that alcohol may cause to the foetus. Women need to be provided with alternative strategies to manage any anxiety and stress. Further, midwives who might be currently promoting the use of alcohol for stress relief also need to be well informed of the potential harms of alcohol in pregnancy and the current NHMRC guidelines [9]. Alcohol should not be recommended as a stress relief method in any population, particularly among pregnant women, and it might benefit midwives and other health professionals to have knowledge of referral pathways for services such as breathing and meditation, Cognitive Behavioural Therapy (CBT), and mindfulness. For example, resources such as MoodGYM are convenient and user-friendly tools that could be referred to by health professionals to help pregnant women who experience stress, anxiety and other mental health problems [27].

It was noted by participants in the study that health professionals, particularly midwives, might encourage the use of wine not only for stress relief, but as a benefit to the developing baby. This may be due to the limited scientific evidence that red wine may reduce incidence of heart disease [28]; however, no research to date has found that alcohol consumption improves the physical health of a developing baby, and it is recommended that pregnant women abstain from all types of alcoholic beverages [9]. It was also discussed among participants that wine would be the most socially acceptable type of alcoholic beverage to consume during a pregnancy, especially in comparison to spirits and hard liquor. This is in keeping with previous research which has suggested that pregnant women were more likely to consume wine as their alcohol of choice [24]. It is therefore important to convey the idea that all alcoholic beverages have the potential to cause harm in an unborn baby, and to ensure that health professionals, pregnant women, and the general public are clear that wine, beer and spirits can be equally harmful.

One of the most important findings of this study was the acknowledgement of the role of the partner in supporting women during pregnancy. Previous research has suggested that the partner may have a significant impact on a woman’s decision to drink during pregnancy, and that encouraging partners to decrease their alcohol use would help to decrease the woman’s alcohol use [18,29,30]. While the findings from the current study did not support the idea that a partner’s drinking habits would influence a woman’s alcohol consumption, it was noted that the partners were still supportive. For the participants in this study it was found that the women were unconcerned whether or not their partner cut back his alcohol use or continued to drink; however, it was important for the partner to provide support for the woman’s decision. This would suggest that future health campaigns do not necessarily need to focus on reducing the alcohol consumption of the partners, but focusing more on ensuring adequate levels of support for women, and an acknowledgement that the decision not to drink may be a difficult choice for pregnant women. From the partner’s point of view, the men in this study were supportive of their partner’s decision not to drink and had carefully considered reducing their own alcohol use.

### Limitations

There are several limitations that should be taken into consideration when interpreting the results of this study. The participants are not a representative sample of the larger population and therefore, the findings are not generalizable. A limitation of convenience sampling is the possibility that there may be an under-representation or over-representation of particular groups of people within the sample. The majority of participants were Caucasian and born in Australia. Therefore, other ethnic and cultural influences need to be taken into consideration. Secondly, due to the nature of focus group discussions there is a potential for bias as participants can sometimes feel peer pressure to provide a socially acceptable response, or may give similar responses.

Finally, due to cancellations one of the focus groups was attended by only 2 participants. This may have resulted in a more restrained discussion than other groups; however, it was agreed to include these participants so that the data generated from this discussion was not lost. It was thought that the responses from two participants would add to the volume of data generated from the other focus groups and would not undermine any of the conclusions drawn. Overall, the findings reflect the knowledge and views of the participants in this study and give an insight into the issues concerning alcohol consumption during pregnancy.

### Implications

The findings from this research provide justification for improving the quality and consistency of information provided to pregnant women about alcohol consumption, as well as improving communication between women and their health professionals. It is clear that consistent, evidence based messages need to be promoted and need to address commonly held misconceptions, particularly around the quantity and timing of foetal alcohol exposure. This study highlights a need for continual discussions about alcohol consumption during pregnancy, which could be undertaken as a standard part of routine clinical practice, such as taking blood pressure or foetal heart rate monitoring at every appointment. Similarly, it is important to ensure that all pregnant women are routinely assessed for alcohol consumption and that the NHMRC guidelines are adhered too. Previous research has indicated that there may be significant barriers preventing health professionals addressing alcohol consumption with pregnant women. These include the assumption that most women do not drink much alcohol during pregnancy and that women already know not to drink, as well as issues such as: alcohol is not on the list of priorities during the antenatal consultation; the burden of consultation is already vast; and the perception that questioning women on their alcohol consumption could appear judgemental and cause anxiety or guilt [15]. It is essential that barriers such as these are addressed, as routine assessment for alcohol consumption in pregnant women needs to be achieved.

This study also confirms the importance of shared decision making, and the role of partners and families in prenatal alcohol consumption. Despite an increase in knowledge and awareness of the adverse effects of alcohol consumption in pregnancy, the prevalence of FASD worldwide does not appear to be decreasing. Thus, while women remain central to FASD prevention, the inclusion of partners and families and a deeper understanding of the societal factors that influence a woman’s drinking may play a key role in tackling this important health issue.

## Conclusions

Findings from this research provide important insights in to the relationship between pregnant women, their partners, and their health care providers in relation to alcohol consumption during pregnancy. This information may be used to develop more appropriate public health messages to improve knowledge about the effects of alcohol consumption during pregnancy among the wider community, as well as targeted groups. Furthermore, it is important to develop strategies that improve communication between health professionals, pregnant women, partners and families. These findings emphasize the need to provide accurate and comprehensive information about the effects of alcohol consumption on the developing baby, particularly with regard to the lack of evidence about safe quantities of alcohol, and the timing of the exposure. In order to improve knowledge on the topic, messages should include clear and consistent advice, and provide alternative options for relieving stress.

These findings emphasize the need to provide ongoing education about alcohol consumption during pregnancy for pregnant women, health professionals, as well as women’s partners, friends, family members and the broader general community. Additionally, the findings highlight the need for more thorough and consistent routine enquiry for alcohol consumption in pregnant women than occurs in current practice. The findings also suggest a lack of evidence-based, up-to-date education among health professionals. Therefore, the need to ensure ongoing Continuing Professional Development (CPD) relating to alcohol consumption in pregnancy is a high priority.
